# Better performance of cerebral blood volume images synthesized from arterial spin labeling and standard MRI in separating glioblastoma recurrence from treatment response than arterial spin labeling

**DOI:** 10.3389/fonc.2025.1647254

**Published:** 2025-09-04

**Authors:** Danyang Wu, Yongsheng Pan, Yunxiao Zhou, Cuiyan Wang, Jingzhen He, Jiaxin Xiang, Bao Wang

**Affiliations:** ^1^ Department of Radiology, Qilu Hospital of Shandong University, Jinan, China; ^2^ School of Computer Science and Engineering, Northwestern Polytechnical University, Xi’an, Shaanxi, China; ^3^ Department of Radiology, Shandong Provincial Hospital Affiliated to Shandong First Medical University, Jinan, China; ^4^ MR Research Collaboration, Siemens Healthineers Ltd., Shanghai, China

**Keywords:** arterial spin labeling, cerebral blood volume, tumor recurrence, glioblastoma, magnetic resonance imaging

## Abstract

Cerebral blood volume (CBV) maps play an important role in the differential diagnosis between glioblastoma recurrence and treatment response. However, it needs high injective velocity. This study aimed to synthesize CBV maps from arterial spin labeling (ASL) and standard MRI sequences using a deep learning method and to validate its discriminating value. A total of 744 MRI scans from 364 patients were included in this retrospective, single-institution study. A three-dimensional (3D) incrementable encoder–decoder network (IEDN) designed for an asymmetrical sample size was trained to synthesize the CBV maps from ASL and the standard MRIs. The synthetic performance was evaluated quantitatively using the structural similarity index (SSIM) and the peak signal-to-noise ratio (PSNR) and qualitatively using a four-point Likert scale (from 0 to 3). In 96 patients suspected of glioblastoma recurrence *vs*. treatment response as the external test set from a hospital-based cohort, the difference in the additive value between synthetic CBV maps and ASL to standard MRIs was examined using the *Z* test. The best algorithm appeared to be achieved by the 3D IEDN with ASL + T1-weighted imaging (T1WI) + T2-weighted imaging (T2WI) + apparent diffusion coefficient (ADC) maps + post-contrast T1WI (SSIM = 88.69 ± 3.97%, PSNR = 32.76 ± 3.39 dB). For the image quality scores, the mean image quality score for all synthetic CBV maps was 2.90. Standard MRI plus synthetic CBV maps had better performance than standard MRI and ASL scans in the differential diagnosis between tumor recurrence and treatment response (*p* = 0.019). Therefore, 3D IEDN produced qualified synthetic CBV maps without the need for high injective velocity from ASL and standard MRIs. The synthetic CBV maps achieved better performance in the differential diagnosis between glioblastoma recurrence and treatment response.

## Introduction

Patients with glioblastoma often fall into a pitfall of confusing tumor recurrence with treatment response after radiotherapy (1, 2). Standard MRI has quite limited values in differentiating recurrence from radionecrosis (3). Cerebral blood volume (CBV) maps may be the most commonly used and the recommended advanced MRI technique for the differential diagnosis between tumor recurrence and treatment response (4). This technique requires a much more exogenous contrast agent and a much higher injective velocity to guarantee an important phenomenon called the “bolus effect.” (5).

In clinical practice, in comparison with the condition in which patients have adverse reactions to the gadolinium contrast agent, a more commonly seen clinical scenario is that patients undergoing radiochemotherapy often have a fragile vessel such that a much higher injective velocity of the contrast agent is prohibited. As the contrast agent could only be injected with a very low velocity, this means that the “bolus effect” for CBV estimation could not be guaranteed. Arterial spin labeling (ASL) is a well-known brain perfusion evaluation technique that does not require exogenous contrast and the bolus injection effect (6, 7). However, ASL often has false-negative results when identifying the nature of the enhancing lesions with a low flow rate (8) and when it encounters vascular variation (9). Therefore, “bolus effect”-independent CBV maps are meaningful for these patients.

Both cerebral blood flow maps derived from ASL (CBF-ASL) and CBV maps derived from dynamic susceptibility contrast-MRI (DSC-MRI) are used for the differential diagnosis between tumor recurrence and treatment response (6, 10). Although CBF and CBV are distinct perfusion metrics, deep learning has demonstrated excellent image translation performance across different MRI modalities (11–13), even MR perfusion modalities (14). Because standard MRI and CBF-ASL do not require the bolus effect, image translation work from standard MRI and CBF-ASL into CBV maps with deep learning appears to be the recommended method for the generation of “bolus effect”-independent CBV maps. However, comparison of the performance of this type of synthetic CBV maps with that of ASL in the differential diagnosis between glioblastoma recurrence and treatment response has yet to be explored.

Therefore, this study aimed to explore the feasibility of synthesizing “bolus effect”-independent CBV maps from ASL and standard MRI scans and to validate its performance in the differential diagnosis between tumor recurrence and treatment response.

## Materials and methods

### Study sample

In this retrospective study, all MRI examinations of brain tumors with quantitative CBV maps and ASL performed between December 2016 and July 2019 were retrieved from the picture archiving and communication system of the Radiology Department. The inclusion criteria were as follows: 1) primary and recurrent primary brain tumors confirmed by pathological results; 2) brain metastases confirmed by pathological results; and 3) follow-up MRIs available for patients in 1) and 2). The exclusion criteria were: 1) compromised quality of the CBV maps; 2) compromised quality of the ASL maps; 3) compromised image quality of the standard MRIs; and 4) patients less than 18 years old.

The data used in this study overlapped those of a previous study that explored the possibility of generating CBV maps from standard MRI scans using a deep learning-based technique (11). This retrospective study was approved by the local ethics committee. All experiments were performed in compliance with the Declaration of Helsinki. As this is a retrospective study, the requirement for informed consent was waived after approval of the local ethics committee.

### Image acquisition and preprocessing

All patients were imaged in the supine position on a 3-T MRI scanner (Magnetom, Skyra; Siemens Healthineers, Forchheim, Germany) using a transmit/receive quadrature 20-channel head-and-neck coil. The imaging protocol was the same for all patients. In our institution, a three-dimensional (3D) ASL with a single post-labeling delay was used. Detailed information on the MRI protocols and the ASL and CBV maps are summarized in **Supplementary Methods 1–3**.

### 3D incrementable encoder–decoder network

In a previous study, the 3D encoder–decoder network showed the best performance in synthesizing quantitative CBV maps (11). This method was used in this study. However, in consideration of the relatively small number of paired ASL and CBV maps, experiments on a small number of subjects may not be able to derive a convincing conclusion. Therefore, an incrementable encoder–decoder network (IEDN) that can learn from many subjects, only a small number of which have ASL images, was proposed. This means that a dataset without ASL, but with standard MRI [including T1-weighted imaging (T1WI), T2-weighted imaging (T2WI), T2-weighted fluid-attenuated inversion recovery imaging (T2-FLAIR), apparent diffusion coefficient (ADC) maps, and post-contrast T1WI] and CBV maps, could be maintained. This specific design contributes to the improvement of the training performance. The input MRI modalities included standard MRI scans and ASL, and 16 types of modality combinations were used. The ground-truth MRI modality comprised the CBV maps.

**Figure 1:**
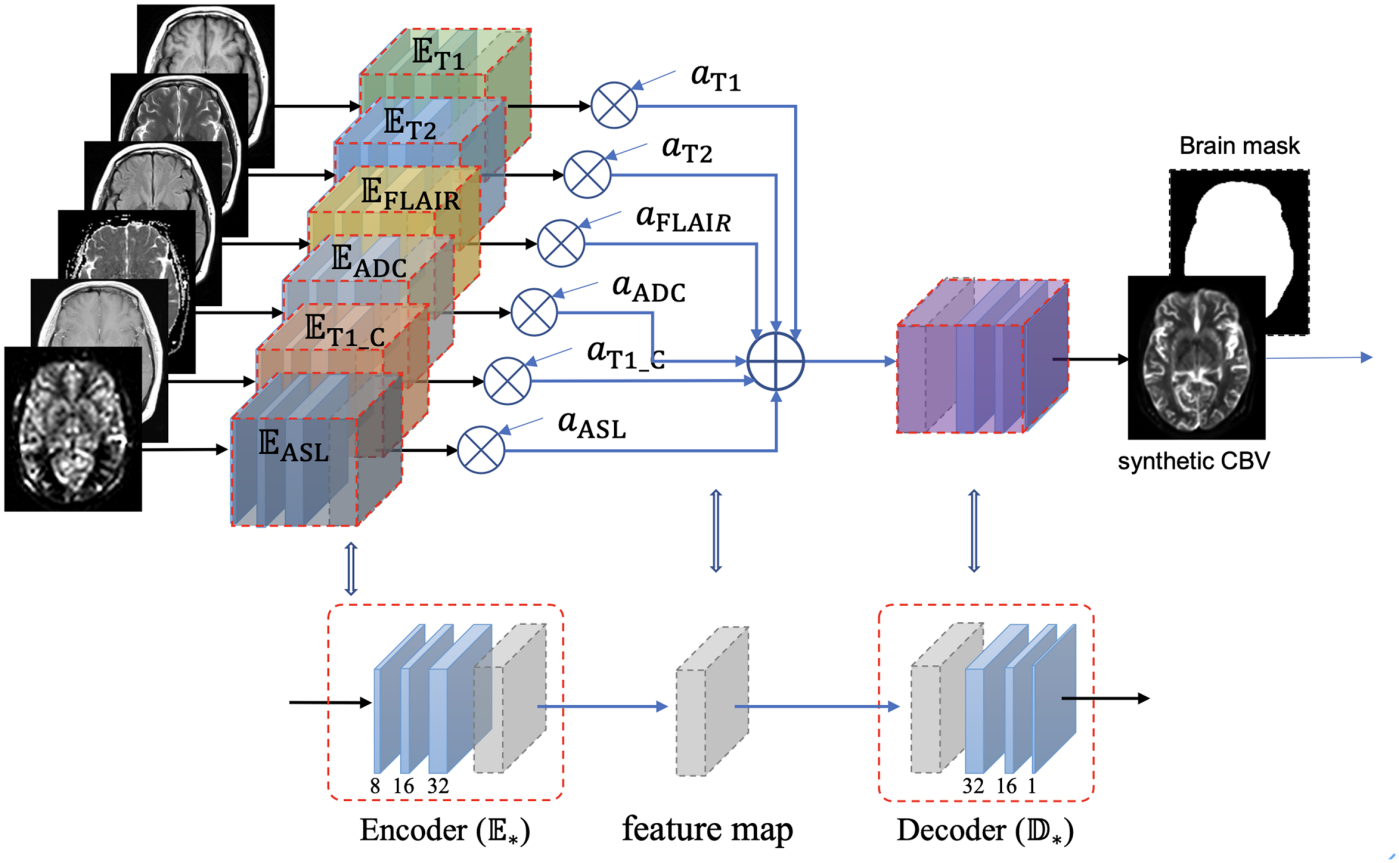
Structure of the incrementable encoder–decoder network (IEDN), which duplicates an encoder part $E_{\text{m}}$ for each modality and calculates a weighted average of the latent feature maps of all encoders before inputting them into the decoder part. A subject with any absent image, $S_{\text{m}}$ , can also be inputted into this network by setting the corresponding weight $a_{\text{m}}$ to 0, thus can be trained or applied to all subjects.


The structure of the IEDN is shown in **Figure 1**, which contains an encoder $E_{\text{m}}$ for each MRI modality and a decoder $D_{\text{CBV}}$ for the CBV modality. The latent feature maps of multiple encoders were averaged into a mixture feature map, which enables the structure to not be affected by the absence of any modality. Denote $S=\{S_{\text{T}1},S_{\text{T}2},\cdots ,S_{\text{ASL}}\}$ as a subject with multiple MRI modalities and $A=\{a_{\text{T}1},a_{\text{T}2},\cdots ,a_{\text{ASL}}\}$ indicates their status of absence ( $a_{\text{m}}=1$ for present and $a_{\text{m}}=0$ for absence, m∈{T1,T2,⋯,ASL} ), its CBV image can be estimated as:

$$S_{\text{CBV}}=D_{\text{CBV}}(\frac{\sum_{\text{m}}\;a_{\text{m}}E_{\text{m}}(S_{\text{m}})}{\sum_{\text{m}}\;a_{\text{m}}})$$

Unlike the previous study that trained a model for each modality combination, this study generally applied one model to any modality combination. Based on the IEDN, we can train a model with ASL using the same subjects as those in our previous study and set $a_{\text{ASL}}$ =0 to simulate a model without ASL. In this study, both the IEDN and the primary 3D encoder–decoder models were used for training.

The MRI scans were randomly split into approximately 3:1 for the training and the test sets. There was no validation set. Instead, the networks were trained constantly for 500 epochs. Multiple scans of the same subjects were aligned to T1-MRI to ensure that they were of the same resolution and voxel spacing. As most of the subjects did not have misalignments between the different scans, only reslicing was performed. The misalignment in several subjects was corrected by linear registration. We used the Adam optimizer with a mean absolute error and set the learning rate to 0.001.

The codes were implemented in Python (version 3.10) with PyTorch2.1 and were uploaded in GitHub (https://github.com/YongshengPan/ASLwMRItoCBV), which require the GNU General Public License (GNU GPL).

### Evaluation of the synthetic “bolus effect”-independent CBV maps from the internal dataset

For quantitative evaluation, the structure similarity index measure (SSIM) and the peak signal-to-noise ratio (PSNR) were used to assess the quality of the synthetic “bolus effect”-independent CBV maps. The range of the SSIM was [0, 1], and a larger value indicates better image quality (15). PSNR > 40 dB indicates very good quality, 30 dB ≤ PSNR < 40 dB indicates good (acceptable) quality, 20 dB ≤ PSNR < 30 dB denotes bad quality, and PSNR < 20 dB indicates very bad (unacceptable) quality. Detailed information on SSIM has been described previously (11). The SSIM and PSNR were calculated over the bounding box of each brain.

Qualitative evaluation of the synthetic CBV maps was based on a four-point Likert scale (0, very poor; 1, poor; 2, good; and 3, very good) using the following criteria: overall perfusion distribution of the lesions, overall perfusion degree of the lesions, and the border and shape of the lesion in contrast to the real CBV maps. The assessments were performed by two neuroradiologists (BW and CW, with 9 and 18 years’ experience in MRI perfusion, respectively). In case of divergence, consensus was reached by these two radiologists after a discussion. The initial scores were also recorded for consistent evaluation.

### Value of the synthetic “bolus effect”-independent CBV maps in differentiating recurrence from treatment response

External datasets containing only the standard MRI scans and ASL were used in this study. Synthetic “bolus effect”-independent CBV maps were generated from the best combination of the training model and modalities. The value of synthetic “bolus effect”-independent CBV maps in the discrimination between tumor recurrence and treatment response was evaluated by two radiologists (BW and XL, with 17 years’ experience). The datasets were from Qilu Hospital of Shandong University (*N* = 96). For diagnostic accuracy, the values of the standard MRI scans plus ASL and the standard MRI scans plus synthetic “bolus effect”-independent CBV maps were compared. Inter-observer agreement was evaluated after an independent image review. In cases of disagreement between the two radiologists, any discrepancies in the diagnosis were resolved by a third fellowship trained radiologist (JH, with 18 years’ experience). Details of the evaluation methods are provided in **Supplementary Method 4**.

### Statistical analysis

A within-group inter-rater reliability statistic (*r*_wg_) was calculated to evaluate the inter-observer agreement regarding the image quality scores. Individual intraclass correlation coefficients (ICCs) with two-way random effects models were used to evaluate the inter-observer agreement regarding the differential diagnosis. The level of agreement was considered very good at a *r*_wg_ or ICC value ≥0.9, good at 0.70–0.89, fair at 0.50–0.69, and poor at ≤0.49. For model performance evaluation, the differences in the SSIM between the two models were compared using a paired *t*-test.

The area under the curve (AUC) for each model (standard MRIs + ASL *vs*. standard MRIs + synthetic “bolus effect”-independent CBV maps) was calculated and compared for statistical significance. Differences were considered statistically significant at a two-sided *p*-value <0.05. Statistical analyses were performed by BW using R version 3.2.0 (www.r-project.org) and MATLAB (version 2023a; MathWorks, Inc., Natick, MA, USA).

## Results

### Study population

**Figure 2:**
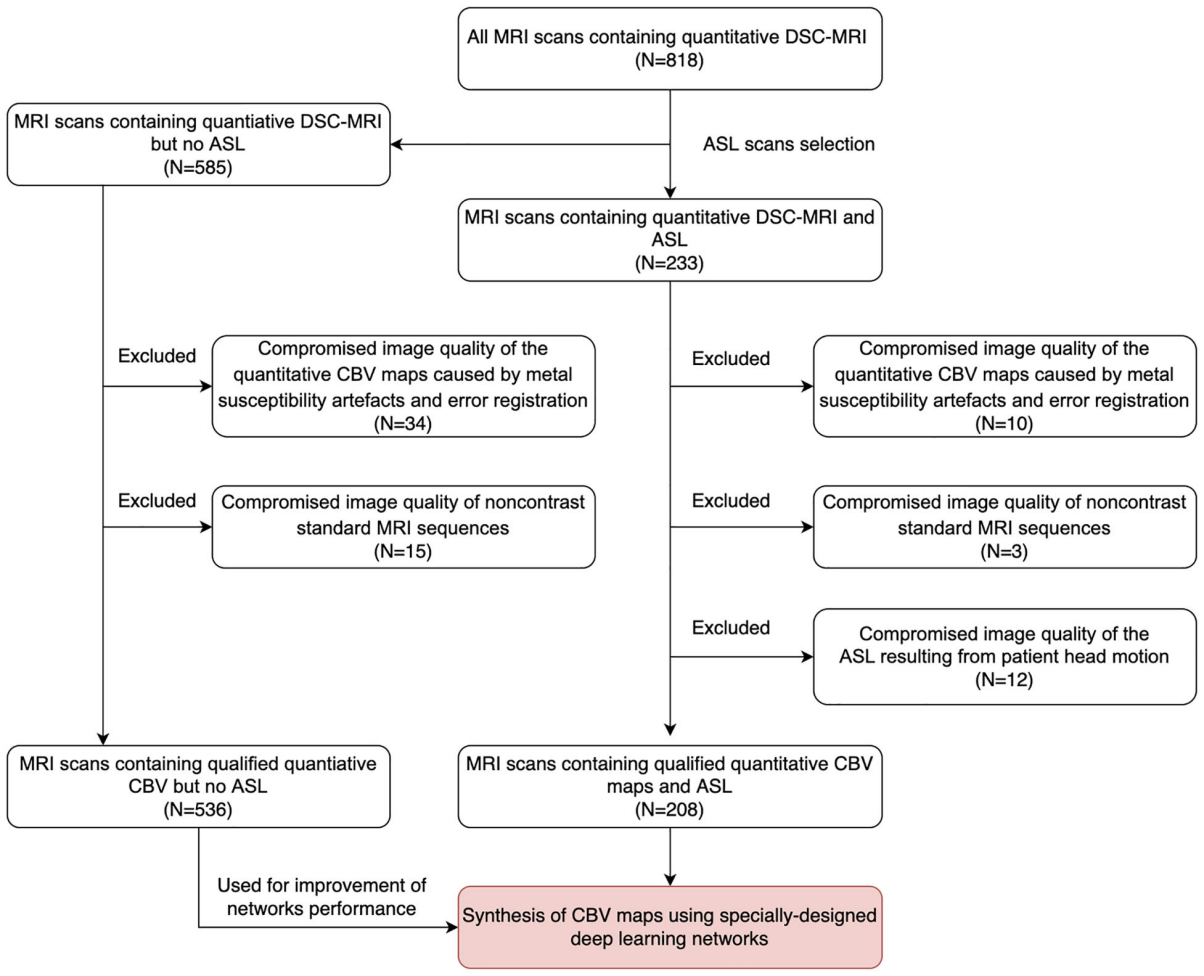
Data collection flowchart. MRI scans for inclusion screening and the final study sample. *DSC*, dynamic susceptibility contrast; *ASL*, arterial spin labeling; *CBV*, cerebral blood volume.

Of the 233 MRI scans with ASL (123 patients, 92 men; mean age = 57.2 ± 9.4 years, range = 18–72 years), 10 (5.3%) were excluded due to the compromised image quality of the quantitative CBV maps caused by metal susceptibility artifacts and error registration, 12 (6.4%) scans were excluded due to the compromised image quality of ASL resulting from patient head motion, and 3 (1.6%) MRI scans were excluded due to the compromised image quality of the standard MRI sequences. Of the 585 MRI scans without ASL, 34 (5.8%) scans were excluded due to the compromised image quality of the CBV maps and 15 (2.6%) scans were excluded due to the compromised image quality of the standard MRI sequences. Finally, 208 MRI scans with ASL from 129 patients (78 men; mean age = 57.0 ± 9.2 years) and 536 MRI scans without ASL from 235 patients (122 men; mean age = 58.9 ± 10.3 years) were included in this study. The study sample is shown in **Figure 2**. Detailed information of the study sample is summarized in **Table 1**.

**Table 1:** Clinical information of the study sample used for image translation training.

No. of patients	364
No. of MRI scans	744
Mean no. of scans per patient (range)	2.12 (1–5)
Mean age (years)	58.53 ± 10.16
Sex	
Men	200 (54.9%)
Women	164 (45.1%)
Type of brain disease	
Glioma	132 (36.3%)
Brain metastasis	180 (49.5%)
Meningioma	16(4.4%)
Lymphoma	12 (3.3%)
Medulloblastoma	14 (3.8%)
Other^a^	10 (2.7%)

Unless otherwise stated, data are presented as the number of patients with percentages in parentheses.

a Including solitary fibrous tumors and germinomas.

### Evaluation of all algorithms with different sequence combinations

**Figure 3:**
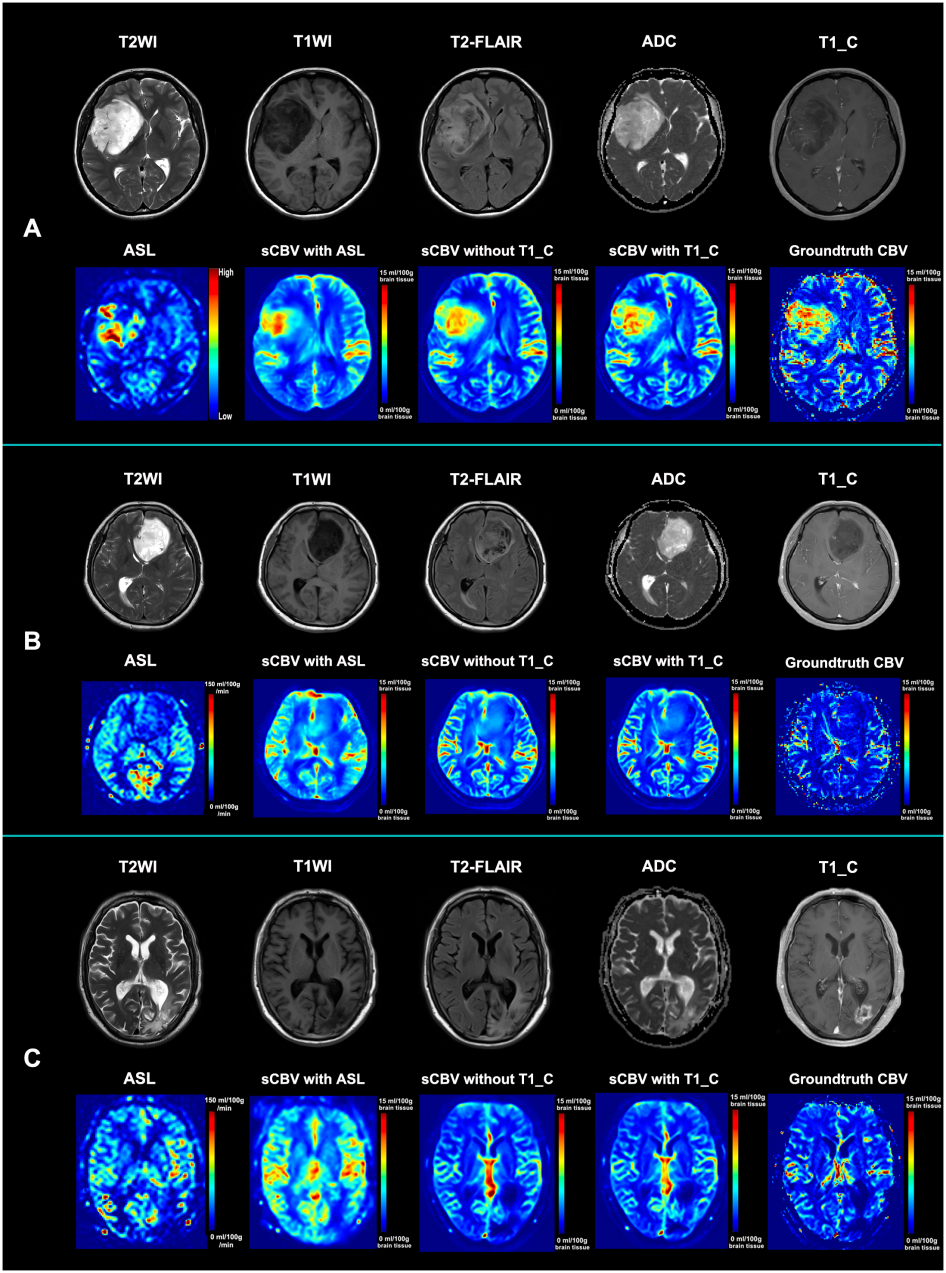
Illustration of the standard MRI, ASL, synthetic CBV maps, and ground-truth CBV maps. **(A)** Standard MRI, ASL, and synthetic CBV and ground-truth CBV maps from a 53-year-old female patient diagnosed with astrocytoma (WHO grade IV). Although the tumor is not obviously enhanced, both the ASL and CBV maps show hypoperfusion within the tumor. Both synthetic CBV maps with or without post-contrast T1WI for synthesis are similar to the ground-truth maps. **(B)** Standard MRI, ASL, and synthetic CBV and ground-truth CBV maps from a 47-year-old male patient diagnosed with astrocytoma (WHO grade II). Although the tumor has a heterogenous signal intensity pattern, both the ASL and CBV maps show hypoperfusion within the tumor. Both synthetic CBV maps with or without post-contrast T1WI for synthesis are similar to the ground-truth map. **(C)** Standard MRI, ASL, and synthetic CBV and ground-truth CBV maps from a 63-year-old female patient 3 months after radio- and chemotherapy with glioblastoma (who was finally diagnosed as an early treatment response). Although the abnormal region is obviously enhanced, both the ASL and CBV maps show hypoperfusion within the region. Both synthetic CBV maps with or without post-contrast T1WI for synthesis are similar to the ground-truth map. *sCBV with ASL* denotes that the synthetic CBV map was generated only from ASL; *sCBV without T1_C* indicates that the synthetic CBV map was generated from ASL, T2WI, T1WI, and ADC maps; *sCBV with T1_C* means that the synthetic CBV map was generated from ASL, T2WI, T1WI, ADC maps, and post-contrast T1WI. *T2-FLAIR*, T2-weighted fluid-attenuated inversion recovery imaging; *ADC*, apparent diffusion coefficient; *ASL*, arterial spin labeling; *sCBV*, synthetic cerebral blood volume map; *T1_C*, post-contrast T1-weighted images.

The results of the 17 generative models for the two algorithms in the validation cohort are summarized in **Table 2**. The best algorithm without post-contrast T1WI appeared to be achieved by IEDN with ASL+T1WI+T2WI+ADC maps (SSIM = 85.52 ± 3.93%, PSNR = 31.43 ± 3.24 dB). The best 3D encoder–decoder algorithm also appeared to be achieved with ASL+T1WI+T2WI+ADC maps (SSIM = 85.33 ± 4.45%, PSNR = 31.19 ± 3.23 dB). The best IEDN algorithm showed a slightly better performance than the 3D encoder–decoder (*p* = 0.003). The algorithm with the worst performance was the 3D encoder–decoder model with ASL only (SSIM = 77.48 ± 4.21%, PSNR = 29.29 ± 2.43 dB). The best algorithm had a significant improvement than that with the ASL-CBF only (*p* < 0.001). Furthermore, the addition of post-contrast T1WI could significantly improve the performance of the best algorithm without post-contrast T1WI (SSIM = 88.69 ± 3.97%, *p* < 0.001; PSNR = 32.76 ± 3.39 dB, *p* < 0.001). Therefore, the best combination of the training model and the MRI modalities was the IDEN model with ASL+T1WI+T2WI+ADC maps+post-contrast T1WI. The standard MRI, ASL, synthetic CBV maps, and ground-truth CBV maps are illustrated in **Figure 3**.

**Table 2:** Results of the 17 generative models of two algorithms from ASL and standard MRI scans.

Sequence	SSIM (%)		PSNR (dB)	
	3D IDEN	3D encoder–decoder	3D IDEN	3D encoder–decoder
ASL-CBF only	78.66 ± 4.91	77.48 ± 4.21	29.44 ± 2.69	29.29 ± 2.43
ASL-CBF+T2	83.54 ± 5.36	82.81 ± 4.17	30.92 ± 3.31	30.20 ± 2.85
ASL-CBF+T1	83.45 ± 4.00	83.15 ± 4.27	30.71 ± 2.90	30.32 ± 2.89
ASL-CBF+T2-FLAIR	83.23 ± 3.84	83.12 ± 4.14	30.70 ± 2.88	30.55 ± 2.93
ASL-CBF+ADC	84.81 ± 3.83	83.88 ± 4.10	30.91 ± 3.05	30.80 ± 3.04
ASL-CBF+T1+T2	84.87 ± 4.30	84.64 ± 4.41	31.08 ± 3.10	30.63 ± 3.01
ASL-CBF+T1+T2-FLAIR	83.94 ± 3.99	83.64 ± 4.41	31.06 ± 3.02	30.73 ± 3.04
ASL-CBF+T1+ADC	85.10 ± 3.77	84.62 ± 4.32	31.16 ± 3.09	30.88 ± 3.09
ASL-CBF+T2-FLAIR+ADC	85.06 ± 3.80	84.50 ± 4.33	31.17 ± 3.12	30.94 ± 3.12
ASL-CBF+T2+T2-FLAIR	84.87 ± 4.32	83.52 ± 4.39	31.10 ± 3.13	30.96 ± 3.12
ASL-CBF+T2+ADC	85.36 ± 4.08	85.09 ± 4.32	31.44 ± 3.31	31.15 ± 3.22
ASL-CBF+T1+T2+T2-FLAIR	85.22 ± 4.17	84.79 ± 4.50	31.14 ± 3.11	31.00 ± 3.15
ASL-CBF+T1+T2+ADC	85.52 ± 3.93	85.33 ± 4.45	31.43 ± 3.24	31.19 ± 3.23
ASL-CBF+T2+T2-FLAIR+ADC	85.43 ± 3.97	85.21 ± 4.45	31.42 ± 3.26	31.10 ± 3.20
ASL-CBF+T1+T2-FLAIR+ADC	85.06 ± 3.81	84.75 ± 4.43	31.18 ± 3.09	30.89 ± 3.11
ASL-CBF+T1+T2+T2-FLAIR+ADC	85.42 ± 3.94	85.31 ± 4.52	31.39 ± 3.21	31.05 ± 3.18
ASL-CBF+T1+T2+ADC+post-contrast T1WI	**88.69 ± 3.97**	**86.68 ± 4.65**	**32.76 ± 3.39**	**32.45 ± 3.45**

*SSIM*, structure similarity index measure; *PSNR*, peak signal-to-noise ratio; *IDEN*, incrementable encoder–decoder network; *ASL-CBF*, cerebral blood flow derived from arterial spin labeling technique; *T2-FLAIR*, T2-weighted fluid-attenuated inversion recovery; *ADC*, apparent diffusion coefficient.

The bold text means the best SSIM values of models without postcontrast T1WI.

### Qualitative evaluation of the synthesized “bolus effect”-independent CBV maps

Synthetic “bolus effect”-independent CBV maps generated from the IEDN with ASL+T1WI+T2WI+ADC maps+post-contrast T1WI were used for qualitative evaluation. The inter-observer agreement for the quality of the synthetic “bolus effect”-independent CBV maps was good (within-group *r* = 0.89). The mean image quality score for all the synthetic “bolus effect”-independent CBV maps was 2.90. For the image quality scores, the percentages of the images given one point (rated as poor quality), two points (good quality), and three points (very good quality) were 1% (2/208), 8.2% (17/208), and 90.8% (189/208), respectively.

### Diagnostic accuracy of the synthetic “bolus effect”-independent CBV maps compared with ASL in differentiating glioblastoma recurrence from treatment response

**Figure 4:**
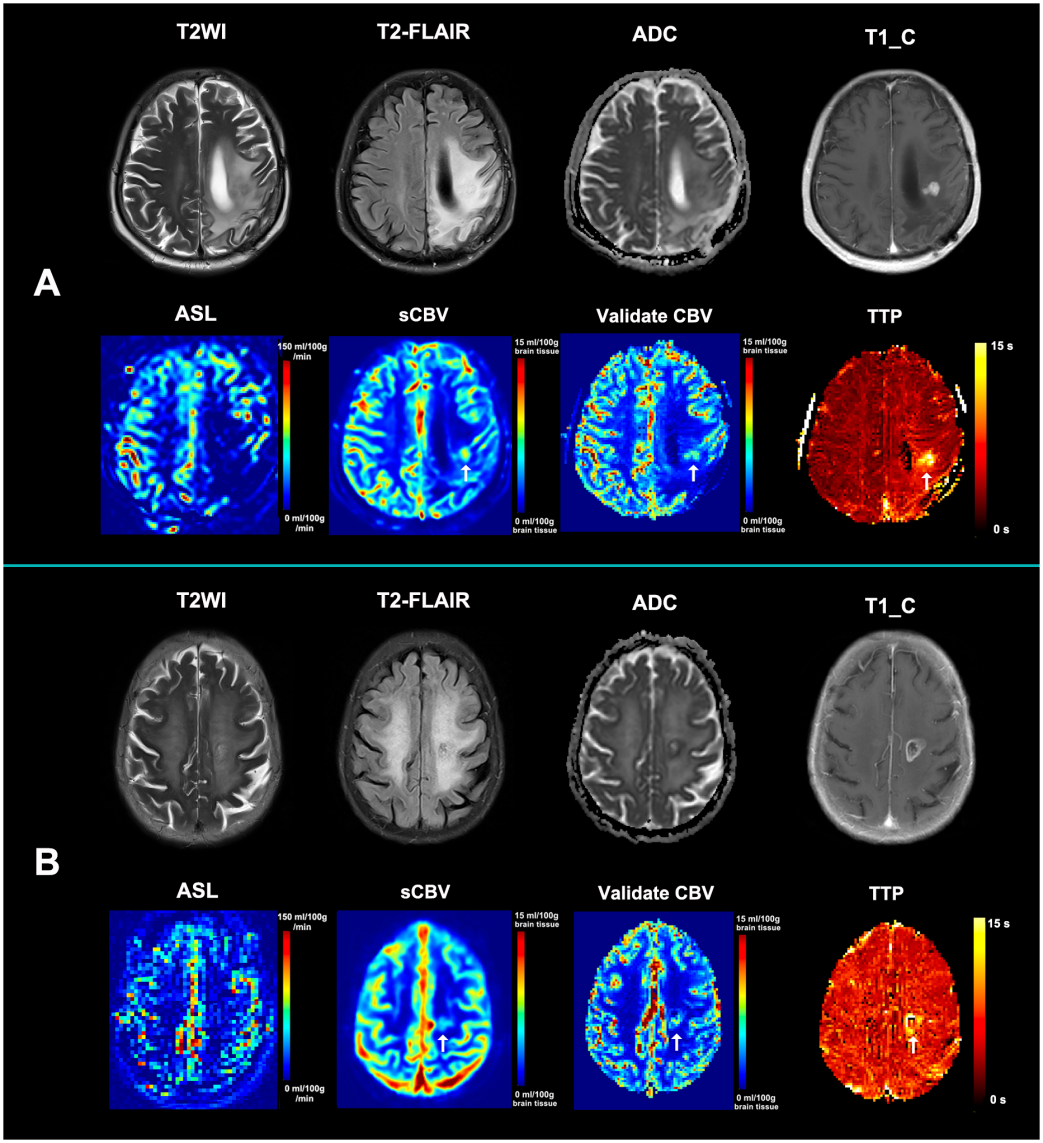
Illustration of the improved accuracy in diagnosing tumor recurrence. **(A)** A new enhancing lesion that appeared in the radiotherapeutic region 3 months after radiochemotherapy in a 65-year-old female patient with glioblastoma. ASL showed hypoperfusion within the enhancing region, while synthetic CBV showed hypoperfusion (*arrow*). A confirmed dynamic susceptibility contrast MRI (DSC-MRI) was then performed, with the relative CBV also demonstrating hypoperfusion the same as the synthetic CBV (*arrow*). The synthetic CBV map correctly recognized the false-negative recurrence lesion in ASL. **(B)** An enlarged enhancing lesion that appeared in the radiotherapeutic region 2 months after radiochemotherapy in a 76-year-old female patient with glioblastoma. ASL showed hypoperfusion within the enhancing region, while synthetic CBV showed hypoperfusion (*arrow*). A confirmed DSC-MRI was then performed, with the relative CBV also demonstrating hypoperfusion the same as the synthetic CBV (*arrow*). The synthetic CBV map correctly recognized the false-negative recurrence lesion in ASL. Both patients were diagnosed with tumor recurrence based on further pathological results and received laser interstitial thermal therapy. The TTP maps from both cases demonstrated an obviously delayed time to peak, indicating that the mismatch between the post-labeling delay time and the real time to peak may be responsible for the underestimated perfusion of ASL. *ASL*, arterial spin labeling; *sCBV*, synthetic non-contrast cerebral blood volume map; *TTP*, time-to-peak map.

Of the 96 patients suspected of glioblastoma recurrence *vs*. treatment response after radiotherapy, 45 (46.9%) had a diagnosis of tumor recurrence and 52 (53.1%) of treatment response. The standard MRIs plus synthetic “bolus effect”-independent CBV maps showed better performance than the standard MRIs plus ASL in differential diagnosis (standard MRIs + ASL: AUC = 0.76, 95%CI = 0.68–0.85; standard MRIs + synthetic “bolus effect”-independent CBV: AUC = 0.87, 95%CI = 0.80–0.94; *p* = 0.019) (**Table 3**), and there was very good agreement between the two evaluations (ICC = 0.84, 95%CI = 0.79–0.88). The improvements were attributed to the improved accuracy in diagnosing tumor recurrence. Illustrations of the improved accuracy in diagnosing tumor recurrence are shown in **Figure 4**.

**Table 3:** Comparison of the performance between standard MRIs plus ASL and synthetic CBV maps in the differential diagnosis of recurrence and TR.

Diagnostic scenario	Standard MRI+ASL			Standard MRI+ synthetic CBV			*Z* test *p*-value
	AUC	Sensitivity	Specificity	AUC	Sensitivity	Specificity	
Recurrence *vs*. TR	0.76 (0.68–0.85)	0.65 (0.52–0.76)	0.89 (0.78–0.95)	0.87 (0.80–0.94)	0.82 (0.71–0.90)	0.88 (0.75–0.94)	0.019

*CBV*, cerebral blood volume; *ASL*, arterial spin labeling; *AUC*, area under the receiver operating characteristic curve; *TR*, treatment response.

## Discussion

Image translation from ASL and standard MRI scans to synthetic “bolus effect”-independent CBV maps is extremely important for patients in whom the qualified “bolus effect” of DSC-MRI could not be guaranteed (4, 5) as the gadolinium-based contrast agent could only be injected with a very low velocity. In this study, it was found that this image translation work is feasible using a deep learning method. The best algorithm was finally achieved with IEDN with ASL+T1WI+T2WI+ADC maps+post-contrast T1WI (SSIM = 88.69 ± 3.97%; PSNR = 32.76 ± 3.39 dB). In addition, standard MRI plus synthetic “bolus effect”-independent CBV maps had better performance than standard MRI plus ASL scans in the differential diagnosis between tumor recurrence and treatment response (*p* = 0.019).

In clinical practice, ASL has great advantages over DSC-MRI among patients with hemorrhage, metal implants, and those with no access to a contrast agent (16). However, its low spatial resolution and low signal-to-noise ratio limit its wider clinical application (7, 16). Although MR machines of higher-strength fields are used to improve the spatial resolution and signal-to-noise ratio of ASL, their quality is not comparable to that of DSC-MRI even from 1.5-T machines (8). Moreover, the results of ASL sometimes require professional interpretation as certain physical and physiological parameters could affect the quality of the ASL image (7, 16). Therefore, generation of perfusion maps with higher spatial resolution and signal-to-noise ratio, but without contrast agent administration, is meaningful in clinical practice.

Although both ASL and CBV maps are perfusion techniques, they vary in terms of perfusion values and patterns, as well as in physiological aspects (8, 17). Our work should be separated from other image translation works that synthesized high-resolution images from low-resolution images (18) (e.g., from low-resolution to high-resolution ASL maps). They are different deep learning works, and they need different paired image data for training.

One of the advantages of our datasets is that quantitative CBV maps (19) paired with ASL maps were used. We considered quantitative CBV maps as being able to maintain the quantitative characteristics of ASL during the model training and to facilitate our quantitative evaluation of the model performance. In addition, the generation of the “bolus effect”-independent CBV maps in this study was both arterial input function-independent and operator-independent (19), and the availability of this type of CBV maps used as ground-truth data may enable a reliable synthesis of “bolus effect”-independent CBV maps.

Our previous study (11) demonstrated that CBV maps could be generated from standard MRI scans, while the image translation work from structural MRIs to advanced MRIs may have a weak biological and technical interpretability. The addition of ASL into the training process of the deep learning network may improve the medical interpretability of the image translation work. Moreover, in our previous study with only standard MRI, the best SSIM of 86.29 ± 4.30 was reached. In this study, after retraining, with the addition of ASL into the training modalities, the best SSIM of 88.69 ± 3.97 was achieved (paired *t*-test: *p* < 0.001). This result emphasizes the additive value of ASL in improving synthetic performance to standard MRI.

The commonly used ASL is often with a single post-labeling delay, which has great influence on its cerebral blood flow maps (16). For the suspected lesions in the deep white matter region, particularly for relatively small lesions in the centrum semiovale, the appropriate pulse labeling delay times vary (8). This may be the reason for the ASL sometimes showing an underestimated perfusion in the suspected lesions. However, the synthetic “bolus effect”-independent CBV maps could help remind observers whether there is an overestimation or an underestimation. In addition, radiologists should pay more attention to ASL-negative patients after radiotherapy.

Synthetic CBV maps from standard MRIs have limited biological interpretability due to their nature of being from structural to functional images. However, the addition of ASL to standard MRI appears more sufficient for radiologists due to both CBF-ASL and CBV maps being brain perfusion techniques. On the other hand, the synthetic process of deep learning methods has to be addressed as it is still unexplored in that the physiological features that play important roles in the whole process are still unknown. This is the core limitation of deep learning. The clinical value of this study is that the synthetic CBV maps could provide more radiological evidence for radiologists whether the true CBV maps cannot be acquired due to patient or machine factors. Satragno et al. (20) found that systemic inflammation markers also demonstrated value in differential diagnosis. The addition of this biomarker information to the synthetic process may further reinforce its biological plausibility and clinical relevance.

This study has several limitations. Firstly, although certain techniques that focus on small samples were used, the training dataset sample was relatively small for the image translation work. Moreover, a number of scans were from the same subjects, which may be transported to the training and test sets separately, and the final results would have been influenced by selective bias. Secondly, the ASL technique with multiple pulse labeling delay times (21) was not used in this study and could not be compared with either ASL with single post-labeling delay or the synthetic CBV maps. Thirdly, based on our clinical experience in differential diagnosis between tumor recurrence and treatment response, the extent, the size, and the location of the enhancing lesions may have an influence on the ASL results. Attention should be paid to the possible selection bias resulting from the study cohort. Fourthly, comparison of the diagnostic performance between the synthetic CBV maps and the actual contrast-enhanced CBV maps was not performed as we did not have enough data for the differential diagnosis between glioblastoma recurrence and treatment response that had both ASL and actual contrast-enhanced CBV maps. Finally, although IEDN was able to handle missing modalities in our CBV synthesis, its generalization needs further validation in common real-world artifacts, variations in the scanner hardware, or patient motion.

In conclusion, we performed an image translation work from ASL maps and standard MRIs to generate synthetic “bolus effect”-independent CBV maps, with the synthetic CBV maps showing better additive value to standard MRIs than ASL in discriminating between tumor recurrence and treatment response in glioblastoma after radiotherapy. However, further studies with larger datasets should be performed to further validate its clinical value.

## Data Availability

The original contributions presented in the study are included in the article/**Supplementary Material**. Further inquiries can be directed to the corresponding author.
