# Supplementary material for: Better performance of cerebral blood volume images synthesized from arterial spin labeling and standard MRI in separating glioblastoma recurrence from treatment response than arterial spin labeling
Source: Front Oncol. 2025 Sep 4;15:1647254. doi: 10.3389/fonc.2025.1647254 (PMC12443552; doi:10.3389/fonc.2025.1647254)
Supplement: Supplementary file 1 [file DataSheet1.docx]

Supplementary Material

**Supplementary Method 1. The detailed information of MR imaging protocols**

All patients were imaged in the supine position on a 3 T MRI scanner (Magnetom, Skyra; Siemens Healthineers) using a transmit/receive quadrature 20-channel head-and-neck coil. The imaging protocol was the same for all patients.

Axial T2-weighted, ASL, precontrast T1-weighted, and T2-FLAIR sequences, and diffusion-weighted imaging (DWI) with b values of 0 s/mm^2^ and 1,000 s/mm^2^ were acquired. Bookend dynamic susceptibility contrast (DSC) perfusion weighted images were acquired after 46 s of injector delay, and then a bolus of 0·2 mmol per kg bodyweight of contrast agent (Gd-DTPA, Magnevist; Schering) was administered, followed by a 20 ml saline flush. An injection velocity of 4·0 ml/s (commonly over 4.5 ml/s) was used. Axial postcontrast T1-weighted (T1_C) was then performed. The slice positions for all the imaging sequences were identical throughout the scans. All 2D MRI sequences had the same imaging scale, position, and slice thickness, which facilitates registration among different modalities. The detailed parameters are as follows:

***T2-weighted imaging (T2WI):*** repetition time (TR): 3700 ms, echo time (TE): 109 ms, slice number: 19, field of view (FOV): 220 mm, slice thickness: 5 mm, distance factor: 30%, flip angle (FA): 150°, voxel size: 0.3×0.3×5.0 mm^3^, accelerate factor: 2, bandwidth: 220 Hz/Px, echo spacing: 9.9 ms.

***Precontrast and postcontrast T1-weighted imaging (T1_C)***: TR: 1820 ms, TE: 13 ms, slice number: 19, FOV: 230 mm, slice thickness: 5 mm, distance factor: 30%, FA: 150°, inversion time (TI): 825 ms, voxel size: 0.4×0.4×5.0 mm^3^, accelerate factor: 2, bandwidth: 260 Hz/Px, echo spacing: 13 ms.

***T2-weighted and fluid-attenuated inversion recovery imaging (T2_F)****:* TR: 8000 ms, TE: 81 ms, slice number: 19, FOV: 220 mm, slice thickness: 5 mm, distance factor: 30%, FA: 150°, inversion time (TI): 2370 ms, voxel size: 0.7×0.7×5.0 mm^3^, accelerate factor: 2, bandwidth: 289 Hz/Px, echo spacing: 9.02 ms.

***Diffusion-weighted imaging (DWI):*** TR: 3700 ms, TE1: 65 ms, TE2: 104 ms, slice number: 19, FOV: 230 mm, slice thickness: 5 mm, distance factor: 30%, FA: 180°, voxel size: 1.4×1.4×5.0 mm^3^, acceleration factor: 2, bandwidth: 919 Hz/Px, echo spacing: 0.36 ms, diffusion directions: 3, diffusion mode: 3-Scan trace, diffusion weighting: 2, noise level: 100, b value: 0 and 1000.

***Postprocessing of apparent diffusion coefficient (ADC) map:*** Centralized data analysis was performed at a single site to derive the ADC from DWI images using a monoexponential fit between pairs of acquired b = 0 and b > 0 s/mm^2^ values. ADC was calculated for three separate DWI directions to characterize each individual gradient channel. The applied diffusion gradient direction in magnet coordinates was derived from the DICOM header (assigned to a specific gradient channel). Information on DWI image postprocessing (e.g. spatial filtering) was not available from the DICOM header but was confirmed by individual sites to exclude optional filtering. To correct for channel-specific b > 0 s/mm^2^ image distortion induced by eddy currents, two-dimensional full-affine co-registration of the b = 0 s/mm^2^ image was performed for DWI data that exhibited substantially different (> 5 mm) phantom tube displacements and/or misshaping for different gradient channels (DWI directions). For systems with significant initial distortions, the co-registration efficiency was assessed by visual inspection of the consistent phantom tube position and shape for all DWI directions for each given image slice prior to ADC map generation. The co-registration of major distortions improved the resulting ADC map uniformity (reduced histogram width) for systems with high eddy current distortions on the selected gradient channels. ADC was calculated on a pixel-by-pixel basis.

**Supplementary Method 2.** **Bookend DSC-PWI and quantitative CBV map**

Scale-PWI, a prototype bookend DSC-PWI sequence provided by Siemens Healthineers, was used in this study. The Scale-PWI sequence merged the pre- and postcontrast T1 mapping into the GRE-EPI sequence for DSC-PWI and added the same “gradient noise” between T1 mapping and the DSC-PWI scan to avoid head motion. The imaging parameters of Scale-PWI were as follows: TR/TE, 1,600 ms/30 ms; bandwidth, 1,748 Hz/pixel; 21 axial slices; field of view (FOV), 220 × 220 mm; voxel size, 1·8×1·8×4 mm^3^; slice thickness, 4·0 mm, and flip angle (FA), 90°. For each slice, 50 measurements were acquired for bookend DSC-PWI analysis.

Quantification of CBV was based on the bookend technique, where the value of absolute CBV is dependent on the change in white matter before and after the injection of the contrast agent. ${CBV}_{WM}=\frac{\left( \frac{1}{T_{1}^{Post}}-\frac{1}{T_{1}^{Pre}} \right)_{WM}}{\left( \frac{1}{T_{1}^{Post}}-\frac{1}{T_{1}^{Pre}} \right)_{Blood}}\times100\%$，quantification of CBV_WM_: $\mathrm{qCBV}_{WM}=WCF\left( \Delta R_{1} \right)\times\frac{1}{\rho}\times\frac{1-{Hct}_{LV}}{1-{Hct}_{SV}}\times{CBV}_{WM}$, where$\mathrm{WCF}\left( \Delta R_{1} \right)=8.2\times{10}^{-3}\Delta R_{1}^{2}+0.25\Delta R_{1}+0.51$，$\rho, {Hct}_{LV,} {Hct}_{SV,} \Delta R_{1}$ are all constant values. The aCBV of each voxel was calculated using the following function: $aCBV=rCBV\times\frac{{qCBV}_{WM}}{{rCBV}_{WM}}$. The unit of absolute CBV is ml/100 g.

**Reference**

**1**. Carroll TJ, Horowitz S, Shin W et al. Quantification of cerebral perfusion using the "bookend technique": an evaluation in CNS tumors. *Magn Reson Imaging*. 2008;26(10):1352-9

**Supplementary Method 3. ASL with a single pulse labeling delay time**

For the ASL acquisition, a prototype pseudo-continuous labelling sequence with background suppression, and a 3D gradient-and-spin-echo (GRASE) readout was used. Additional sequence parameters: labelling duration was 1800ms, with a 1500ms post-labelling delay, turbo factor=12, EPI factor=31, segments=2 (with parallel imaging, GRAPPA=2), repetitions = 10; acquisition time = 5 min 9 s (3 T). A proton-density weighted (M0) image was also acquired (TR=4000ms), with identical readout to the ASL acquisition but with the labelling RF pulses removed, for CBF quantification. To minimize artefacts from through-plane blurring inherent in the 3D GRASE sequence, the echo train length used for the pCASL sequences was kept below 300 ms.

**Supplementary Method 4.** **Validation methods for the** **value of synthetic CBV maps in clinical practice**

For comparison between tumor recurrence and treatment response, the evaluation methods were the same as those mentioned above. The accuracy of the two classification methods (standard MRIs + ASL *vs*. standard MRIs + synthetic “bolus effect”-independent CBV maps) was explored and compared using the chi-squared test. Recurrent glioma was finally diagnosed based on the histological results of stereotactic brain biopsy (22/49) or craniotomy (27/49). Treatment response was diagnosed using the histological results from craniotomy operation (8/47), stereotactic biopsy (15/47), or follow-up (14/47) results (based on Response Assessment in Neuro-Oncology for glioma [***Ref 1***], in which complete response, partial response, and stable diseases were all considered as treatment responses).

Treatment response is generally indistinguishable from recurrence on conventional structural MRI. Two neuroradiologists reviewed the conventional MRs and tried to separate each enhancing region into tumor recurrence and treatment response. A consensus was reached on the conventional MRIs of each enhancing lesion based on the following criteria: radiation modality, radiation dose and volume, interval time since the last radiotherapy, use of concurrent and/or adjuvant chemotherapy, enhancing region related to the original tumor location or paracele, enhancing pattern with a “Swiss cheese” or “soap bubble,” hemorrhage, necrosis, and degree of edema [***Ref 2***]. When they reviewed the perfusion maps, hyperperfusion (defined as higher blood perfusion than normal-appearing white matter) within the abnormal region indicated tumor recurrence.

**Ref 1**. Wen PY, Macdonald DR, Reardon DA, et al. Updated Response Assessment Criteria for High-Grade Gliomas: Response Assessment in Neuro-Oncology Working Group. J Clin Oncol. 2010;28(11):1963-1972. doi:10.1200/JCO.2009.26.3541

**Ref 2**. Winter SF, Loebel F, Loeffler J, et al. Treatment-induced brain tissue necrosis: a clinical challenge in neuro-oncology. Neuro Oncol. 2019;21(9):1118-1130. doi:10.1093/neuonc/ noz04
